# Impact of peritoneal cytology on survival of endometrial cancer patients treated with surgery and radiotherapy

**DOI:** 10.1038/sj.bjc.6601446

**Published:** 2003-11-25

**Authors:** P M Tebeu, G Y Popowski, H M Verkooijen, J Casals, F Lüdicke, G Zeciri, M Usel, C Bouchardy, A L Major

**Affiliations:** 1 Department of Obstetrics and Gynaecology, Geneva University Hospitals, Switzerland; 2 Department of Radiation Oncology, Geneva University Hospitals, Switzerland; 3 Geneva Cancer Registry, Institute for Social and Preventive Medicine, Geneva University, Switzerland; 4 Fondation pour Recherches Médicales, University of Geneva, Switzerland; 5 Department of Obstetrics and Gynaecology, Yaounde University Hospitals, Cameroon

**Keywords:** endometrial cancer, peritoneal cytology; survival, radiotherapy

## Abstract

Stage IIIA endometrial cancer includes patients with serosal or adnexal invasion and patients with positive peritoneal cytology only. In this study, we assessed the impact of peritoneal cytology on endometrial cancer survival. All endometrial cancer patients receiving surgery and radiotherapy at the Geneva University Hospitals between 1980 and 1993 were included. Stage lllA cancers were categorised into ‘cytological’ stage lllA (only positive peritoneal cytology) and ‘histological’ stage lllA (serosal or adnexal infiltration). Survival rates were analysed by Kaplan–Meier method and compared using log-rank test. The prognostic importance of peritoneal cytology was analysed by multivariate regression analysis. This study included 170 endometrial cancers (112 stage I, 17 cytological stage IIIA, 18 histological stage IIIA, 9 stage lllB+). Disease-specific survival of cytological stage IIIA was not different from stage I (94 *vs* 88% respectively, *P*=0.5) but better than histological stage IIIA (94 *vs* 51% respectively, *P*<0.01). Histological stage IIIA patients were at increased risk to die from cancer compared to stage I patients (HR 2.7, 95% CI 1.0–7.7), while cytological stage IIIA patients were not (HR 0.3, 95% CI 0.3–2.0). Cytological stage lllA endometrial cancer has similar prognosis as stage l and better prognosis than histological stage IIIA. Additional research, definitively separating stage and cytology is warranted.

Endometrial cancer is the most common genital tract malignancy in Western countries (Grady and Ernster, 1996; Burke *et al*, 1997; Parkin *et al*, 1997; Greenlee *et al*, 2000). The long-term survival of patients with endometrial cancer is related to the stage at diagnosis. Disease-specific 5-year survival for tumours confined to the uterus is over 80%, but decreases to approximately 40% for tumours invading the serosa, the fallopian tube or the ovary (AJCC, 2002).

Before 1988, endometrial cancer was staged clinically (International Federation of Gynaecology & Obstetrics, FIGO 1971). This staging system was based on the fractional biopsy of the endometrium, the size of the uterine cavity and physical examination. The clinical system was abandoned because newly accumulated data from surgical staging reports, among others including depth of myometrial invasion, allowed stratification. A new surgical staging system was approved at the 1988 FIGO meeting (Grady and Ernster, 1996; Burke *et al*., 1997; FIGO, 2000; AJCC, 2002). In addition to pathological extension, the result of cytology obtained by peritoneal washings became an important determinant of staging. In particular, stage l patients with positive peritoneal cytology were upstaged to stage lllA (Konski *et al*, 1988). Stage IIIA therefore covers both patients with only positive peritoneal cytology and patients with macroscopic/histological invasion of serosa or adnexal tissues.

As some studies have suggested that cytology is an important prognostic factor, while others did not (Grimshaw *et al*, 1990; Morrow *et al*, 1991; Kadar *et al*, 1992; Obermair *et al*, 2001; Preyer *et al*, 2002; Kasamatsu *et al*, 2003), the value of peritoneal cytology in the staging of endometrial cancer is still controversial (Kasamatsu *et al*, 2003). In this study, we evaluated the impact of positive peritoneal cytology on the survival of patients operated for endometrial carcinoma and treated with adjuvant brachytherapy and/or external radiotherapy.

## METHODS

### Patients

The current study included women who received surgery followed by adjuvant external radiotherapy and/or brachytherapy for endometrial cancer between 1980 and 1993 at the departments of Gynaecology and Radiation Oncology of the Geneva University Hospitals (*n*=295). We excluded patients with stage I or stage II endometrial cancer without peritoneal cytology assessment (*n*=111), women with previous malignancy occurring within 5 years prior to the diagnosis of endometrial cancer (*n*=2), or patients who were not resident in the canton (*n*=12). The final study population included 170 patients. According to the Geneva cancer registry, these women represented approximately 50% of all endometrial cancer patients treated in the public sector during the period considered.

Peritoneal washing was performed by either collecting liquid present in the peritoneal cavity, or by rinsing the cavity with 100 cm^3^ of physiological saline. The liquid was centrifuged and assessed for the presence of malignant cells. Surgical treatment generally included hysterectomy and oophorosalpingectomy, except for three patients (<2%) who received only simple hysterectomy. A total of 12 patients (7%) underwent lymph node dissection. All surgical procedures were followed by external radiotherapy and/or brachytherapy.

### Variables

Histologic type and differentiation of the endometrial tumours were coded according to the International Classification of Diseases for Oncology (World Health Organization, 1990). A patient was considered to have positive peritoneal cytology if adenocarcinoma cells were detected, regardless of the number of cancer cells. Stages were coded according to the 1988 FIGO staging system: stage I, tumour confined to uterus; stage II, tumour invading cervix; stage IIIA, tumour associated with positive peritoneal cytology or macroscopic or histological involvement of serosa or adnexa and stage IIIB+, tumour invading vagina, mucosa of bladder/bowel, regional lymph node or distant metastases. For the purpose of the present study, stage lllA cancers were further categorised as: ‘cytological’ stage lllA defined as cases with only positive peritoneal cytology and ‘histological’ stage lllA defined as cases with histological or macroscopic infiltration of serosa or adnexal tissues.

Other variables of interest were: age at diagnosis (<50, 50–69, ⩾70 years), period of diagnosis (1980–87, 1988–93), differentiation (good, moderate, poor, unknown), degree of myometrial invasion (<50%, ⩾50%), type of surgery (hysterectomy and oophorosalpingectomy with and without lymphadenectomy), and type of radiotherapy (external with brachytherapy, external only, brachytherapy only).

Data on survival and follow-up were derived from the Geneva cancer registry and included vital status and date of death or departure from the canton (regularly and systematically obtained from the Cantonal Population Office) and cause of death (retrieved from medical files).

### Statistical analyses

The 5-year overall and disease-specific survival rates were calculated. Disease-specific survival was calculated considering death from endometrial cancer only. Survival curves were obtained by Kaplan–Meier method and were compared by nonparametric survival analyses using log-rank test. The effect of peritoneal cytology on endometrial cancer mortality was analysed by multivariate Cox's proportional-hazards regression analysis, taking into account other variables significantly linked to survival. Analyses were performed using SPSS (Hull and Nie, 1995). Differences were considered statistically significant at *P*<0.05.

## RESULTS

Of the 170 endometrial cancer patients included in the study, 112 were diagnosed as stage I, 14 as stage II, 17 as cytological stage IIIA, 18 as histological stage IIIA, and nine were diagnosed as stage IIIB+. If peritoneal cytology had not been incorporated in the staging of the endometrial cancers, 15 of the 17 cytological stage IIIA tumours would have been classified as stage I and two as stage II.

Table 1

**Table 1:** Characteristics of 170 endometrial cancer patients according to stage

	**Stage I (*n*=112) *n* (%)**	**Stage II (*n*=14) *n* (%)**	**Stage IIIA cyt (*n*=17) *n* (%)**	**Stage IIIA hist (*n*=18) *n* (%)**	**Stage IIIB+(*n*=9) *n* (%)**
Mean age (range) years	64 (33–81)	66 (55–78)	66 (51–81)	65 (42–90)	65 (37–77)
					
*Age category (years)*					
<50	7 (6)	—	—	2 (11)	1 (11)
50–69	71 (63)	10 (71)	12 (71)	10 (56)	4 (44)
70+	34 (31)	4 (29)	5 (29)	6 (33)	4 (44)
					
*Period of diagnosis*					
1980–1987	65 (58)	4 (29)	5 (29)	5 (28)	7 (78)
1988–1993	47 (42)	10 (71)	12 (71)	13 (72)	2 (22)
					
*Invasion of myometrium*					
<50%	70 (63)	4 (29)	8 (47)	5 (28)	4 (44)
⩾50%	42 (37)	10 (71)	9 (53)	13 (72)	5 (56)
					
*Differentiation*					
Good	67 (60)	5 (45)	8 (57)	6 (38)	2 (22)
Moderate	30 (27)	4 (36)	3 (21)	4 (25)	4 (44)
Poor	15 (13)	2 (18)	3 (21)	6 (38)	3 (33)
Unknown	—	3	3	2	—
					
*Surgical treatment*					
Hysterectomy	3 (3)	—	—	—	—
Hysterectomy+unilat/Bilateral annexectomy Idem plus lymphadenectomy	105 (94)	13 (93)	16 (94)	17 (94)	4 (44)
	4 (3)	1 (7)	1 (6)	1 (6)	5 (56)
					
*Radiotherapy*					
External and brachy	46 (42)	11 (92)	11 (58)	15 (83)	7 (78)
External only	8 (7)	—	3 (21)	3 (17)	2 (22)
Brachy only	56 (51)	1 (8)	3 (21)	—	—
Unknown	2	2	—	—	—

Stage I=tumour confined to uterus; stage II=tumour invading cervix; stage IIIA cyt=positive peritoneal cytology; stage IIIA hist=histological involvement of serosa or adnexa; stage IIIB+=tumour invading vagina, mucosa of bladder/bowel, regional lymph node or distant metastases.

presents the characteristics of these 170 patients according to stage of endometrial cancer. The mean age of the patients was 64.7 years and was similar for the different stages. Compared to histological stage IIIA tumours, cytological stage IIIA tumours invaded the myometrium to a lesser extent, were better differentiated and received less aggressive radiotherapy.

The median duration of follow-up was 126 months and 14 patients (8%) were lost to follow-up. Table 2

**Table 2:** Observed and disease-specific 5-year survival after endometrial cancer according to stage

	***n***	**Number of endometrial cancer deaths**	**Median follow-up (days)**	**Overall 5-year survival^a^ (%)**	**Disease specific 5-year survival^b^ (%)**
Stage I	112	14	1825	85	88
Stage II	14	8	1176	36	43
Stage III cyt	17	1	1825	94	94
Stage IIIA hist	18	8	1048	44	51
Stage IIIB +	9	5	1224	44	44

**Figure 1:**
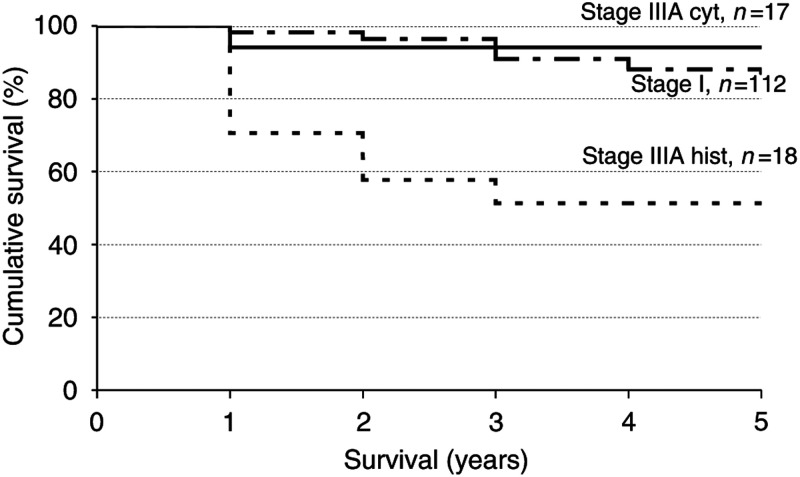
Disease-specific survival curves of endometrial cancer for the stages I, cytological IIIA and histological IIIA. Stage I=tumour confined to uterus; stage IIIA cyt=positive peritoneal cytology; stage IIIA hist=histological involvement of serosa or adnexa. Log-rank test of comparison of survival of stage I *vs* cytological stage IIIA: *P*=0.5; log-rank test of comparison of survival of cytological stage IIIA *vs* stage histological IIIA: *P*=0.008.

Stage I=tumour confined to uterus; stage II=tumour invading cervix; stage IIIA cyt=positive peritoneal cytology; Stage IIIA hist=histological involvement of serosa or adnexa; stage IIIB+=tumour invading vagina, mucosa of bladder/bowel, regional lymph node or distant metastases.

a All cases of death.

b Death from endometrial cancer.

presents overall and disease-specific 5-year survival rates per stage. Figure 1

presents disease-specific survival curves for stage I, cytological stage IIIA and histological stage IIIA endometrial cancer. The disease specific 5-year survival of patients with cytological stage IIIA tumours was not significantly different from that of patients with stage I tumours (94 *vs* 88% respectively, log-rank test: *P*=0.5). In contrast, the 5-year disease-specific survival of patients with histological stage IIIA tumours was significantly worse (51 *vs* 94% for histological *vs* cytological stage IIIA cancers, log-rank test: *P*<0.01). A similar pattern was observed when considering the overall survival rates accounting for all deaths, including death from other causes than endometrial cancer.

After adjustment for factors significantly linked to survival from endometrial cancer (i.e. age, tumour differentiation and type of radiotherapy) as shown in Table 3

**Table 3:** Hazard ratio^a^ of death from endometrial cancer according to stage

	**HR^a^**	**95% CI**
Stage I	1^b^	
Stage II	3.2*	1.2–8.6
Stage IIIA cytological	0.3	0.3–2.0
Stage IIIA histological	2.7^c^	1.0–7.7
Stage IIIB and more	3.5*	1.2–10.4

a Hazard ratio are derived from Cox model adjusted for age, tumour differentiation and type of radiotherapy (external radiation therapy, brachytherapy or both).

b reference category.

c *P* borderline significant (0.057).

* *P*<0.05. Stage I=tumour confined to uterus; stage II=tumour invading cervix; stage IIIA cyt=positive peritoneal cytology; Stage IIIA hist=histological involvement of serosa or adnexa; stage IIIB+=tumour invading vagina, mucosa of bladder/bowel, regional lymph node or distant metastases.

, the risk to die from endometrial cancer was still not significantly different in patients with cytological stage IIIA compared with patients with stage I cancer (HR 0.3, 95% CI 0.3–2.0). Compared to stage I, patients with stage II, histological IIIA, and IIIB+ tumours were at respectively 3.2-fold (95% CI 1.2–8.6), 2.7-fold (95% CI 1.0–7.7) and 3.5-fold (95% CI 1.2–10.4) increased risk to die from their endometrial cancer. The results were not modified when excluding the two patients in the cytological stage IIIA category who had cervical invasion (i.e. who would have been staged as stage II in the absence of positive peritoneal cytology).

## DISCUSSION

This study is the first to compare the prognosis of patients with stage I, cytological stage IIIA, and histological stage IIIA endometrial cancer in the same patient population. It suggests that positive peritoneal cytology is not an independent prognostic factor in patients with endometrial cancer treated with surgery and radiotherapy. Irradiated women with only positive cytology (cytological stage IIIA) had the same prognosis as women with endometrial cancer confined to the uterus (stage I) and a much better prognosis than women with histological stage IIIA cancer.

However, our study has several limitations: 111 women had no peritoneal cytology and were therefore excluded, patients were selected for inclusion in the study on the basis of administration of radiotherapy, and the study has a relatively low statistical power due to the limited number of patients included. Nonetheless, the lack of significant survival difference between the stage I and cytological stage IIIA groups suggest that the FIGO classification system classifies patients with positive peritoneal cytology into an inappropriately high-risk category. However, this finding might also be explained by a disproportionate benefit derived from radiotherapy in patients with positive peritoneal cytology.

Table 4

**Table 4:** Overview of studies on survival in women with stage l, cytological stage lllA and histological stage lllA endometrial cancer

	**Survival (%)**				
**Study**	**Stage I**	**Stage IIIA cyt**	**Stage IIIA hist**	***P*-value**	**Remarks**
Preyer *et al* (2002)	—	64^a^	38^a^	=0.05	Exact number of patients with cytological stage IIIA receiving RT unclear. Estimated that less than 50% received ext RT or LND
Morrow *et al* (1991)	93^a^	65^a^	—	<0.05	No routine LND. Exact number of cyt stage IIIA patients treated with RT unclear. Estimated that less than 50% received ext RT
Obermair *et al* (2001)	96^c^	67^c^	—	<0.05	No routine LND. Exact number of patients with cyt stage IIIA receiving RT unclear. Estimated that two of 13 received ext RT
Kasamatsu *et al* (2003)	94^c^	90^c^	—	NS	All patients underwent LND
Kadar *et al* (1992)	93^b^*	88^b^*	—	NS	All patients underwent LND
Tebeu *et al* (this study)	88^b^	94^b^	51^b^	<0.05	80% of patients with cyt stage IIIA received ext RT

a 5-year disease-free survival.

b 5-year disease-specific survival.

c 3-year disease-free survival. LND=lymph node dissection; ext RT=external radiotherapy.

* Estimated from the survival curve.

shows that similarly high survival rates (88–94% 3–5 year survival) in women with stage I and cytological stage IIIA cancer have been observed in series where, as in our study, the large majority of cytological stage IIIA patients underwent either pelvic radiotherapy or total pelvic lymphadenectomy (Kadar *et al*, 1992; Kasamatsu *et al*, 2003). In contrast, the relatively poor results reported by other investigators (64–67% 3–5 year survival) in cytological stage IIIA patients might be explained by the fact that in these studies, only a minority of patients received treatment to pelvic lymph nodes (Morrow *et al*, 1991; Obermair *et al*, 2001; Preyer *et al*, 2002). Pelvic radiotherapy or lymphadenectomy might be especially important for patients with positive peritoneal cytology, as it has been shown by Morrow *et al* (1991) that the presence of positive peritoneal cytology increases the risk of para-aortic lymph node invasion (15 *vs* 4%), and by inference increases the likelihood of pelvic node involvement.

The proportion of cytological stage IIIA cancer patients with cervical involvement is unknown in the majority of studies, except for the study of Kasamatsu *et al* and ours, in which the proportions of cytological stage IIIA patients with cervical involvement were relatively low (0 and 12%, respectively). As cervical involvement is known to affect the survival negatively, a higher proportion of cytological stage IIIA patients with cervical involvement might explain unfavourable survival rates. Therefore, the unknown proportions of patients with cervical involvement complicate interpretation of outcomes and impair comparison between the different studies.

In conclusion, this study found comparable survival rates for patients with cytological IIIA and stage I endometrial cancer, which were significantly superior to that of patients with histological stage IIIA endometrial cancer. Because all studies, including ours, have important shortcomings, no definitive conclusion on the importance of positive peritoneal cytology can be drawn and therefore, additional research is warranted. We strongly recommend that stage and cytology be separated in future studies, in order to better define the impact of peritoneal cytology on outcome of endometrial cancer.
